# Pediatric Hearts and Minds: Reimagining Health Education Through Play and Narrative

**DOI:** 10.2196/72675

**Published:** 2025-03-13

**Authors:** Alina Yang

**Affiliations:** 1 Scarsdale High School, 1057 Post Road, Scarsdale, NY, United States

**Keywords:** congenital heart disease, children health literacy, health education, health education interventions, patient-centered care, design, pediatric, PRISMA

As a student advocate actively involved in heart health promotion among youth, I was interested in the study entitled “Exploring Health Educational Interventions for Children With Congenital Heart Disease: Scoping Review” [1]. The authors identified a gap in age-appropriate educational tools for younger children in the interventions, highlighting the importance of using playful, developmentally tailored strategies to engage them in learning about their cardiac condition.

This insight dovetails precisely with the broader realm of pediatric health literacy, wherein the convergence of cognitive development and medical comprehension calls to attention unique barriers. Children with congenital heart disease (CHD) face difficulties in understanding abstract medical concepts and their condition [2]. Considering this, narrative medicine holds great potential in increasing the relatability, digestibility, and applicability of knowledge by reframing biomedical concepts into metaphorical storytelling. Thus, children with CHD may be able to internalize their medical journey in ways that align with their developmental stage and personal experiences, gaining a sense of agency and coherence.

Similarly, the role of ludic and entertaining pedagogical tools emerges as a pivotal mediator of developmental trajectories across cognitive, social, and emotional domains. Health care providers, especially pediatricians and family physicians, must actively ensure that play is healthy and safe [3]. Whether it be a story-driven game or a cardiac-themed toy, interactive tools may significantly advance educational interventions through tangible learning modalities that convert abstract principles into concrete experiences. As such, the cardiac health journey becomes one of engagement, where the child becomes a conscious, active participant in their medical education rather than a mere recipient of information.

The teddy bear hospital concept in particular exemplifies this symbiotic relationship between play and education, demonstrating efficacy in reducing children’s health care–related anxiety, improving their health care knowledge, and enhancing their well-being through playful role-playing in a health care setting [4]. By softening the edges of medical procedures, this model dismantles the intimidating walls of the clinical environment, transforming it into a less foreign and more approachable space for younger children who are not as impacted by traditional educational interventions.

Yet another crucial dimension of children’s play surfaces through physical activity. Children with CHD and their parents recognize the importance of physical activity, but uncertainty in their health environment contributes to inactivity despite minimal professional restrictions [5]. An area ripe for intervention, physical activity guidelines could incorporate real-time feedback mechanisms that build confidence and ensure safety to promote physical exercise as an act of healing and empowerment, rather than a source of anxiety.

In tailoring educational messaging by age, we can raise the effectiveness of CHD interventions, recognizing and respecting that children are not simply “mini teenagers” and much less “mini adults.” Such an approach must, therefore, incorporate elements of embodied cognition, experiential learning, and psychological support to create a comprehensive educational framework that addresses the specific cognitive and emotional needs of children with CHD. Only in this way can we create immersive and engaging learning environments that make complex cardiac concepts accessible to the young minds often underrepresented while fostering psychological resilience and physical confidence.

## Abbreviations

CHD: congenital heart disease
